# The Predictive Value of N-Terminal Probrain Natriuretic Peptide for Infection in Patients With Acute Myocardial Infarction

**DOI:** 10.3389/fcvm.2021.626724

**Published:** 2021-08-25

**Authors:** YiNing Dai, XiaoLiang Wan, Can Liu, ChongYang Duan, Shuai Shao, HongHuan Chen, Litao Wang, JiJin Lin, Ling Xue, JiYan Chen, PengCheng He, YuanHui Liu, Ning Tan

**Affiliations:** ^1^Department of Cardiology, Guangdong Cardiovascular Institute, Guangdong Provincial Key Laboratory of Coronary Heart Disease Prevention, Guangdong Provincial People's Hospital, Guangdong Academy of Medical Sciences, Guangzhou, China; ^2^Department of Cardiology, Foshan Second People's Hospital, Foshan, Guangdong, China; ^3^Department of Biostatistics, School of Public Health, Southern Medical University, Guangzhou, China

**Keywords:** acute myocardial infarction, percutaneous coronary intervention, N-terminal probrain natriuretic peptide, infection, predictor

## Abstract

**Background:** Infections increase the risk of poor outcomes in patients with ST-elevation myocardial infarction (STEMI) undergoing percutaneous coronary intervention (PCI). However, predicting patients at a high risk of developing infection remains unclear. Moreover, the value of N-terminal probrain natriuretic peptide (NT-proBNP) for predicting infection is still unknown. Thus, we aimed to assess the relationship between NT-proBNP and the following development of infection, and clinical adverse outcomes in patients with STEMI undergoing PCI.

**Methods:** STEMI patients undergoing PCI were consecutively enrolled from January 2010 to July 2016 and divided into groups according to baseline NT-proBNP levels: tertiles T1 (<988 pg/mL), T2 (988–3520 pg/mL), and T3 (≥3520 pg/mL). The primary endpoint was infection during hospitalization.

**Results:** A total of 182 (27%) patients developed in-hospital infection. The incidence of infection increased from T1 to T3 (10.5, 17.7, and 54.5%, *P* < 0.001). NT-proBNP was an independent risk factor (adjusted odds ratio = 1.39, 95% confidence interval (CI) = 1.12–1.73, *P* = 0.003) and presented accurately predicting infection (area under curve = 0.774). Multivariate cox analysis showed that NT-proBNP was a significant risk factor for major adverse clinical events (MACE) at follow-up (adjusted HR = 1.92, 95% CI = 1.61–2.29, *P* < 0.001).

**Conclusion:** The baseline NT-proBNP level has a good predictive value for infection and MACE in STEMI patients undergoing PCI.

## Introduction

Infection is an important complication in patients with ST-elevation myocardial infarction (STEMI) undergoing percutaneous coronary intervention (PCI). It increases the mortality even by 10-fold in 30 days (1–3). Early identification high-risk patients for post-acute myocardial infarction (P-AMI) infections, helps to determine a better treatment strategy, avoided aggressive invasive operations, and potentially improve outcomes.

The N-terminal probrain natriuretic peptide (NT-proBNP) is released predominantly from the ventricular myocardium in response to increased ventricular wall stress (4). It is a well-recognized prognostic marker of multiple disease states, including heart failure (5) and chronic obstructive pulmonary disease (COPD) (6–8), which are potentially associated with a high risk of infection. Previous researches focused on the predictive value of NT-proBNP for clinical outcomes in existing sepsis or septic shock that expanding the clinically awareness and utility of NT-proBNP in these infectious stage (9–11). However, the value of NT-proBNP in predicting infection is still lack of knowledge, especially in patients with AMI. Thus, we aim to assess the relationship between the NT-proBNP and infection, and clinical adverse outcomes in patients with STEMI undergoing PCI.

## Materials and Methods

### Study Population

**Figure 1:**
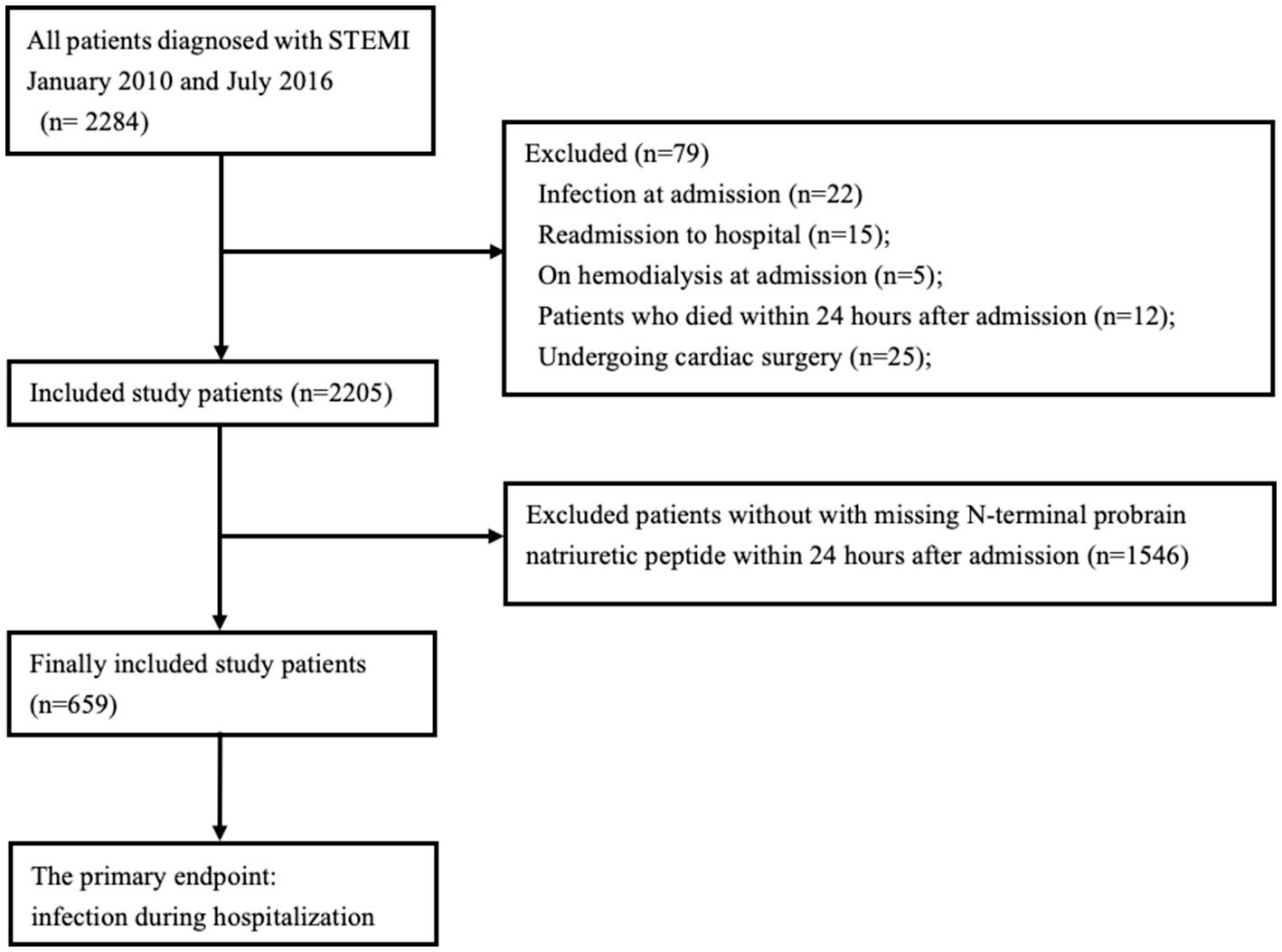
Flow diagram of the study.

Consecutive patients with STEMI undergoing PCI were prospectively enrolled at Guangdong Provincial People's Hospital between January 2010 and July 2016. The diagnosis of STEMI was according to the international guidelines (12). Patients with NT-proBNP measurements within 24 h after hospital admission were enrolled. We excluded the patients who considered as infection, chronic or tumors inflammatory diseases and chronic renal failure required hemodialysis therapy at admission. In addition, we excluded patients who died within 24 h after admission and those undergoing cardiac surgery. The study flow was showed in Figure 1. The study was approved by the research ethics committee of Guangdong Provincial People's Hospital and followed the guidelines stipulated in the Declaration of Helsinki and the ethical standards of the responsible committee on human experimentation. Informed consent was obtained from all included patients.

### Clinical Procedure

NT-proBNP was measured using an electrochemiluminescence immunoassay (Roche Diagnostics, Germany). Furthermore, laboratory tests, such electrolytes, blood lipid, creatinine, and other clinical routine parameter examinations, were also performed within 24 h after admission. Coronary angiography and then PCI were performed according to the guidelines for catheter techniques (13). The choice of stent and need for intra-aortic balloon pump (IABP) were at the discretion of the interventional cardiologist. Antiplatelet therapy as well as other medications were prescribed at the physicians' discretion. Clinical parameters were first collected by one researcher and later confirmed by another one.

### Endpoints and Definitions

The primary endpoint was infection, which was defined as infection requiring antibiotics (14). In addition, infections were categorized as pulmonary, urinary infections, or others (including abdominal sepsis, primary bacteremia, and unidentified primary infection site), by the cardiologist. Secondary endpoints were regarded as in-hospital and follow-up major adverse clinical events (MACE), which were defined as all-cause mortality, stroke, and any bleeding. Our in-hospital adverse clinical events were accumulated by two independent researchers according to the electronic records. After discharge, all of the patients were follow-up by researchers or nurses, through telephone interviews or clinic visits for at least three years.

### Statistical Analyses

Baseline NT-proBNP level was skewed distribution, thus, they were logarithmically transformed (lgNT-proBNP) and sort the orders, then the patients were divided into the following tertiles: T1 (*n* = 219, <988 pg/mL), T2 (*n* = 220, 988–3520 pg/mL), and T3 (*n* = 220, ≥3520 pg/mL). Continuous data were presented as mean ± standard deviation, otherwise data were presented as median and interquartile range and compared using Kruskal-Wallis test. Categorical data were presented as percentages, and χ^2^ test or Fisher's exact test was selected for comparison. Clinical events were compared between patients with and without infection. Receiver operating characteristic (ROC) curves were conducted and the area under the curve (AUC) was then calculated to evaluate the predictive value of NT-proBNP for infection. The optimal statistical cutoff point was selected by using the Youden index (Youden = sensitivity + specificity – 1). Univariate and multivariate logistic regression were used to determine the risk factors for in-hospital infection. Clinically important or significant potential confounders in the univariate analysis were included for multivariable analysis. We performed two multivariate analysis models to adjust the potential risk factor. In model one, we included variables of lgNT-proBNP, sex, smoke, chronic obstructive pulmonary disease, diabetes, stroke, hypertension, prior myocardial infarction, femoral access, serum albumin and white blood cell. In other model, we included the variables of lgNT-proBNP, age, aspirin, contrast volume, clopidogrel, hemoglobin A1c, low-density lipoprotein cholesterol, total cholesterol and statins. The adjusted odds ratio (OR) or adjusted hazard ratio (HR) and 95% confidence interval (CI) were also presented. Univariate analyses of follow-up mortality and MACE were performed using the Kaplan-Meier survival method for patients categorized by NT-proBNP. Multivariate cox regression analysis was also conducted to recognize NT-proBNP for follow-up MACE. SAS version 9.4 (SAS Institute, Cary, NC) software were used to perform the statistical analyses. *P* values were two-sided, and *P* < 0.05 was regarded as significant.

## Results

A total of 659 patients were included (mean age: 64 ± 12 years; female, 23.2%). The baseline characteristics are shown in Table 1. Patients in T3 were older, more likely to be a smoker, had higher Killip grade and longer hospital stay, and tend to have a history of hypertension, stroke, diabetes and COPD. A positive association in heart rate, anemia and creatinine levels were noted with increasing NT-proBNP levels, but a negative association in LVEF, estimated glomerular filtration rate, and transradial access was found.

**Table 1:** Baseline characteristics according to NT-proBNP tertiles.

	**NT-proBNP**			
**Variables**	**T1 (*n* = 219)**	**T2 (*n* = 220)**	**T3 (*n* = 220)**	***P*-value**
Age				
Age>75 year, n (%)	65 (29.7%)	102 (46.4%)	161 (73.2%)	<0.001
Mean (SD), year	58.76 ± 11.90	62.15 ± 12.29	70.73 ± 9.86	<0.001
Female, n (%)	26 (11.9%)	44 (20.0%)	83 (37.7%)	<0.001
Hypertension, n (%)	92 (42.0%)	114 (51.8%)	152 (69.1%)	<0.001
Diabetes, n (%)	48 (21.9%)	57 (25.9%)	92 (41.8%)	<0.001
Hyperlipaemia, n (%)	26 (11.9%)	13 (5.9%)	10 (4.5%)	0.008
Smoking, n (%)	109 (49.8%)	104 (47.3%)	46 (20.9%)	<0.001
COPD, n (%)	1 (0.5%)	3 (1.4%)	18 (8.2%)	<0.001
Prior myocardial infarction, n (%)	6 (2.7%)	11 (5.0%)	17 (7.7%)	0.061
Prior stroke, n (%)	7 (3.2%)	19 (8.6%)	35 (15.9%)	<0.001
Atrial fibrillation, n (%)	5 (2.3%)	7 (3.2%)	14 (6.4%)	0.070
Systolic blood pressure (mmHg)	122.45 ± 22.01	121.93 ± 21.68	121.50 ± 27.77	0.918
Diastolic blood pressure (mmHg)	74.16 ± 12.80	72.36 ± 12.68	71.71 ± 15.28	0.153
Heart rate, beat per minutes	77.06 ± 14.76	82.31 ± 16.19	89.53 ± 21.39	<0.001
Killip class ≥ II, n (%)	44 (20.1%)	70 (31.8%)	151 (68.7%)	<0.001
White blood cell	11.52 ± 3.62	11.45 ± 3.62	12.85 ± 4.71	<0.001
Total cholesterol (mmol/L)	5.13 ± 1.32	4.62 ± 1.15	4.52 ± 1.31	<0.001
LDL-C (mmol/L)	3.30 ± 1.02	2.92 ± 0.98	2.78 ± 1.06	<0.001
HDL-C (mmol/L)	1.00 ± 0.23	0.98 ± 0.26	0.97 ± 0.30	0.515
Anemia, n (%)	53 (24.2%)	84 (38.2%)	137 (62.3%)	<0.001
HbA1c (%)	6.49 ± 1.49	6.49 ± 1.46	6.93 ± 1.68	0.010
Serum albumin (g/L)	34.99 ± 4.00	32.66 ± 4.19	30.48 ± 4.91	<0.001
Hemoglobin (g/L)	136.87 ± 20.41	128.33 ± 28.88	119.31 ± 21.18	<0.001
eGFR (mL/min/1.73 m^2^)	97.92 ± 28.86	82.75 ± 27.00	51.84 ± 28.57	<0.001
Serum creatinine (mg/dL)	82.10 ± 21.08	96.93 ± 48.30	174.73 ± 170.87	<0.001
LVEF (%)	55.38 ± 10.37	51.14 ± 10.04	44.18 ± 12.00	<0.001
Aspirin, n (%)	210 (95.9%)	211 (95.9%)	203 (92.3%)	0.147
Clopidogrel, n (%)	209 (95.4%)	216 (98.2%)	207 (94.1%)	0.088
Statins, n (%)	212 (96.8%)	218 (99.1%)	207 (94.1%)	0.014
ACEI, n (%)	173 (79.0%)	162 (73.6%)	144 (65.5%)	0.006
ARB, n (%)	28 (12.8%)	35 (15.9%)	43 (19.5%)	0.155
CCB, n (%)	16 (7.3%)	26 (11.8%)	35 (15.9%)	0.019
Transradial access, n (%)	193 (90.6%)	167 (84.3%)	126 (69.2%)	<0.001
Stents, median (Q25~Q75)	1 (1~2)	1 (1~2)	1 (1~2)	0.371
Contrast volume ≥ 100 ml, n (%)	145 (70.0%)	149 (78.0%)	122 (69.7%)	0.121
Multi-lesion, n (%)	157 (73.4%)	153 (77.7%)	152 (83.5%)	0.052
Length of hospitalization, median (Q25~Q75)	6 (5~8)	7 (6~10)	10 (6~17)	<0.001

*COPD, chronic obstructive pulmonary disease; LDL-C, low-density lipoprotein cholesterol; HDL-C, high density lipoprotein cholesterol; HbA1c, hemoglobin A1c; eGFR, estimated glomerular filtration rate; LVEF, left ventricular ejection fraction; ACEI/ARB, Angiotensin-Converting Enzyme Inhibitors/Angiotensin receptor blocker; CCB, calcium channel blockers*.

**Figure 2:**
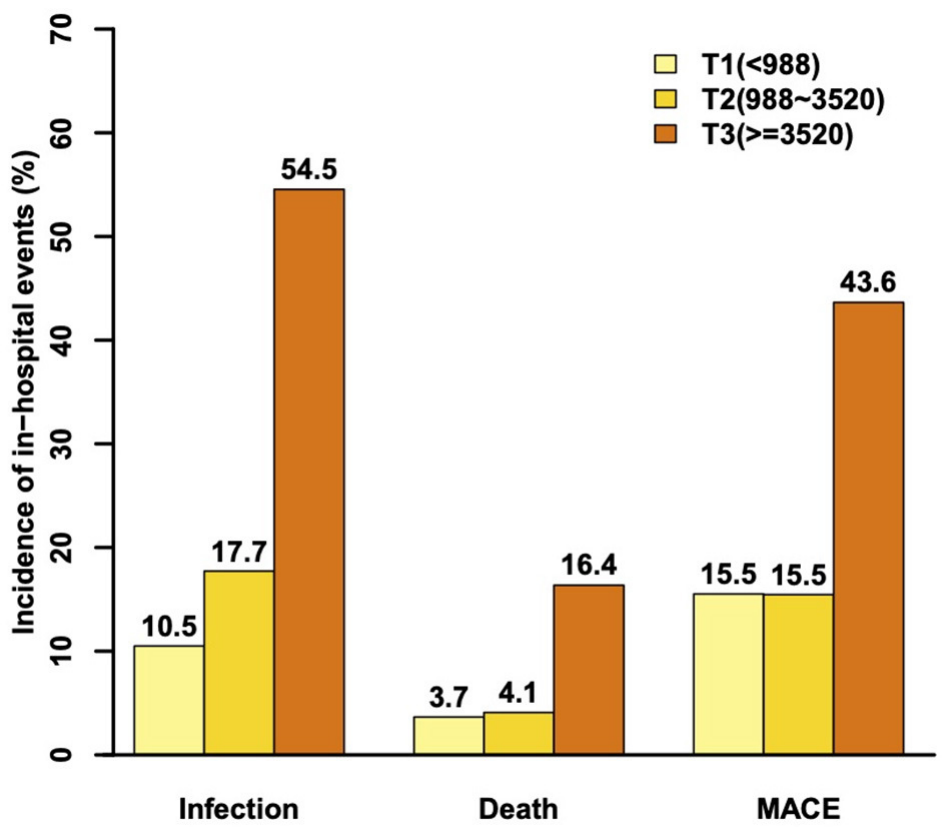
The incidence of infection, death, and major adverse clinical events during hospitalization.

The incidence of infection (10.5, 17.7, 54.5%) and in hospital all cause death (3.7, 4.1, 16.4%) was higher when NT-proBNP increased from T1 to T3 (Figure 2). T1 and T2 have same rates of MACE (15.5% for each) and both lower than that in T3 (43.6%). Patients with infection were more likely to have invasive procedures such as IABP, and adverse clinical events including death, and MACE. In addition, the baseline characteristics of patients with or without infection were compared (Supplementary Table 1). The summary of subtypes of infection were summarized (Supplementary Table 2).

Univariate analysis showed that lgNT-proBNP (OR = 2.16, 95% CI = 1.85–2.53, *P* < 0.001) was associated with infection. After adjusting for other significant factors, the multiple logistic analysis verified that lgNT-proBNP was still a significant risk factor of infection (adjusted OR = 1.39, 95% CI = 1.12–1.73, *P* = 0.003, or adjusted OR = 2.16, 95% CI = 1.73–2.70, *P* < 0.001) (Table 2).

**Table 2:** Independent risk factor of infection based on multivariate analysis.

**Variables**	**OR**	**95% CI**	***P*-Value**
lgNT-proBNP	1.39	1.12–1.73	0.003
Sex	0.54	0.28–1.01	0.054
Smoke	0.61	0.36–1.04	0.069
COPD	2.07	0.56–7.67	0.276
Diabetes	1.20	0.71–2.04	0.494
Stroke	1.82	0.84–3.97	0.131
Hypertension	1.00	0.60–1.67	0.991
Prior myocardial infarction	2.27	0.88–5.88	0.091
Femoral access	2.41	1.35–4.32	0.003
Serum albumin	0.93	0.88–0.98	0.008
White blood cell	1.19	1.12–1.27	0.000

*COPD, chronic obstructive pulmonary disease. lgNT-proBNP (adjusted OR = 2.16, 95% CI = 1.73–2.70, P < 0.001), adjusted for age, aspirin, contrast volume, clopidogrel, hemoglobin A1c, low-density lipoprotein cholesterol, total cholesterol, statins*.

**Figure 3:**
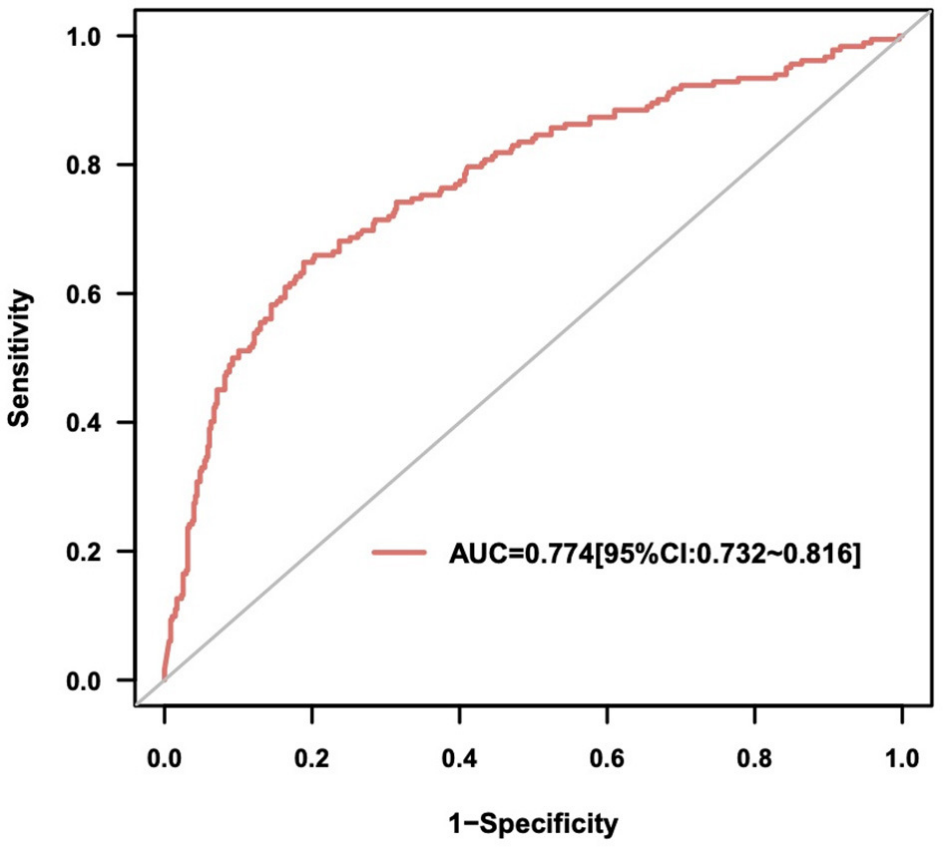
The receiver operating characteristic curve of N-terminal probrain natriuretic peptide (NT-proBNP) for infection.

ROC curve analysis confirmed that NT-proBNP could predict in-hospital infection accurately (AUC = 0.774, 95% CI = 0.73–0.82, *P* < 0.001) (Figure 3). The optimal statistical cutoff point was 3864 pg/mL by using the Youden index. The predictive value of NT-proBNP were evaluated based on the types of infection. The result showed that NT-proBNP had good predictive value for pulmonary infections (AUC = 0.729, 95% CI = 0.63–0.83, *P* < 0.001) and urinary infections (AUC = 0.741, 95% CI = 0.58–0.90, *P* = 0.004) (Table 3).

**Table 3:** NT-proBNP for predicting infections.

**Cutoff = 3864 pg/mL**	**Infection**	**Pulmonary infection**	**Urinary infection**
Sensitivity	0.648	0.669	0.600
Specificity	0.811	0.781	0.696
Positive predictive value	0.567	0.457	0.072
Negative predictive value	0.858	0.896	0.978
Positive likelihood ratios	3.436	3.061	1.971
Negative likelihood ratios	0.433	0.424	0.575
Youden index	0.460	0.450	0.296
Area under curve	0.774	0.729	0.741

Multiple logistic analysis showed that lgNT-proBNP was a risk factor for MACE during hospitalization (OR = 1.61, 95% CI = 1.35–1.92, *P* < 0.001). Kaplan-Meier survival curves showed that patients in higher tertiles had worse outcomes (log-rank test, *P* < 0.001). At follow-up, multivariate cox analysis found that lgNT-proBNP was a risk factor for MACE (adjusted hazard ratio = 1.92, 95% CI = 1.61–2.29, *P* < 0.001).

## Discussion

To the best of our knowledge, our study was the first to show that NT-proBNP was a good predictor for P-AMI infection in patients with STEMI undergoing PCI, and provided additional information on the association between NT-proBNP and infection. In addition, the current study also further confirmed that NT-proBNP was a hospital and follow-up prognostic factor for these patients.

Although the definition of infection in our study was similar to that of a previous study (1), the incidence of infection was higher in our patients than previous studies (1, 2, 15). The difference in results may be due to the following reasons. First, the risk of infection was proved to increase along with aging (15). Given the different methods of data presentation, it was difficult for us to compare previous studies, but our data showed that the higher number of elderly patients in our study might have caused the high incidence of infection. Second, apart from aging, other risk factors of infection, including accompanying diseases, might later increase the risk of invasive procedures. Patients in our study might have worse baseline conditions, as 16.5% of our patients had Killip grades of III-IV, which was higher than those in other studies (1, 2). Moreover, previous studies have reported that the risk of infection increases with the increasing duration of intensive care unit (ICU) stay (16, 17). Another study showed that the median ICU stay was 1 day in stable patients with STEMI treated in the ICU after primary PCI. Thus, the higher incidence of infection in our study may be due to the prolonged median ICU stay, compared to that of a previous study (4 days vs. 1 day) (18).

Whether NT-proBNP or BNP could predict an infection remained unclear. Two previous studies including patients who underwent lung cancer surgery demonstrated that preoperative BNP levels could predict postoperative complications (19, 20), including pneumonia, and a trend of BNP for predicting postoperative pneumonia was observed. The following other two researches also demonstrated that NT-proBNP or BNP could predict later infections. One study by Bobik et al. (21) showed that preoperative NT-proBNP levels could predict postoperative infection in 31 patients with atrioventricular septal defect. The other study showed that BNP could predict urosepsis after ureteroscopy in patients with unilateral ureteral obstruction (22). However, Attaran and colleagues who investigated 141 patients undergoing cardiac surgery showed that the preoperative BNP level was not significantly higher in patients with existing postoperative infection (23). Despite the differences in the design and the great heterogeneity among these studies, most of them were limited by the sample size; thus, a definite conclusion on whether NT-proBNP could predict infection during hospitalization cannot be made in their studies. Besides, an observational study found that NT-proBNP were able to predict (AUC = 0.72) the concurrent infection at the time of admission in patients with AMI, but in our study, such cohort were excluded (3). This study combined with our findings suggested that NT-proBNP might well predict infection in patients with AMI.

It is well known that NT-proBNP measurements are commonly performed in patients with AMI, and our results strengthen the role of NT-proBNP for these patients and call for an earlier test. The current study highlighted that patients with elevated NT-proBNP levels are at a high risk of developing infection. However, the pathophysiologic mechanisms behind their association remained unclear. First, NT-proBNP was proved to be associated with COPD severity and could predict its exacerbation in both patients with and without cardiovascular diseases (6). Strong connections have also been found between COPD and pneumonia, because they share similar risk factors and present overlap in terms of epidemiology (24). Therefore, these connections supported the predictive role of NT-proBNP for pneumonia. However, we should also notice that NT-proBNP remains similar for predicting infection, after adjusting for COPD. There are other unknown mechanisms beside COPD. Second, patients with high NT-proBNP levels were more likely to suffer from cardiovascular events and death (25, 26), which may increase the use of invasive procedures, such as IABP implantation or mechanical ventilation, subsequently increasing the risk of infection (27, 28). Third, the increasing NT-proBNP levels was a marker that reflecting cardiopulmonary stress, including systolic dysfunction, diastolic dysfunction, pulmonary hypertension and right heart strain (29, 30), might cause a large infarction size or serious damage, which would then later increase the risk of developing pneumonia (31).

There were several limitations in our study. First, it was prospective observational study with small sample size, and some of the baseline characteristics was difference between the NT-proBNP groups. Although multivariate analysis was used to reduce the inherent bias, potential confounders due to unevaluated variables were still possible. The inherent limitations of observational study warrant a well-designed, prospective, multi-centers researches to evaluate the relationship between the level of NT-proBNP and clinical outcomes. Second, we did not monitor the dynamic change of NT-proBNP levels; hence, its dynamic effect on P-AMI infection is unknown. Therefore, further exploration is necessary to identify the clinical impact of risk stratification using the dynamic changes of NT-proBNP for infection. Third, given that procalcitonin measurement is expensive and not routinely used in patients with AMI, we were not sure about its predictive value for infection in our study cohort. Fourth, the reverse causality was still possible. Finally, we only included the patients with STEMI; thus, the predictive value of NT-proBNP for infection in other populations should be demonstrated in future investigations.

In conclusion, The NT-proBNP level is an independent predictor for infection in STEMI patients undergoing PCI. Patients with a high level of NT-proBNP should be considered to undergo effective prophylactic strategies for infection.

## Data Availability Statement

The raw data supporting the conclusions of this article will be made available by the authors, without undue reservation.

## Ethics Statement

The studies involving human participants were reviewed and approved by the research ethics committee of Guangdong Provincial People's Hospital. The patients/participants provided their written informed consent to participate in this study.

## Author Contributions

NT and YL: supervisors of the study and guarantee the study data and accuracy. NT and YL: study concept and design. YD and XW: drafting of the manuscript. CD and YL: statistical analysis. All authors acquisition, analysis, interpretation of data, critical revision, and final approval of the manuscript. All authors agreed to submit the manuscript for publication.

## Conflict of Interest

The authors declare that the research was conducted in the absence of any commercial or financial relationships that could be construed as a potential conflict of interest.

## Publisher's Note

All claims expressed in this article are solely those of the authors and do not necessarily represent those of their affiliated organizations, or those of the publisher, the editors and the reviewers. Any product that may be evaluated in this article, or claim that may be made by its manufacturer, is not guaranteed or endorsed by the publisher.
