# Supplementary material for: The Predictive Value of N-Terminal Probrain Natriuretic Peptide for Infection in Patients With Acute Myocardial Infarction
Source: Front Cardiovasc Med. 2021 Aug 25;8:626724. doi: 10.3389/fcvm.2021.626724 (PMC8424116; doi:10.3389/fcvm.2021.626724)
Supplement: Supplementary file 1 [file Table_1.docx]

**Supplementary Appendix 1**

**Table S1. Baseline characteristics in patients with or without infection**

**Table S2. Subtypes of infection according to NT-proBNP tertiles**

**Table S1. Baseline characteristics in patients with or without infection**

|  |  |  |  |
| --- | --- | --- | --- |
| Variables | Infection  (n=182) | Non-infection  (n=477) | P-values |
| Age |  |  |  |
| Age>75 year, n(%) | 121 (66.5%) | 207 (43.4%) | <0.001 |
| Mean (SD), year | 68.14±12.05 | 62.27±12.23 | <0.001 |
| Gender, n(%) |  |  | 0.236 |
| Male | 134 (73.6%) | 372 (78.0%) |  |
| Female, | 48 (26.4%) | 105 (22.0%) |  |
| Hypertension, n(%) | 116 (63.7%) | 242 (50.7%) | 0.003 |
| Diabetes, n(%) | 75 (41.2%) | 122 (25.6%) | <0.001 |
| Hyperlipaemia, n(%) | 9 (4.9%) | 40 (8.4%) | 0.132 |
| Smoking, n(%) | 31 (17.0%) | 30 (6.3%) | <0.001 |
| COPD, n(%) | 15 (8.2%) | 7 (1.5%) | <0.001 |
| Prior myocardial infarction, n(%) | 14 (7.7%) | 20 (4.2%) | 0.069 |
| Prior Stroke, n (%) | 31(17.0%) | 30 (6.3%) | <0.001 |
| Atrial fibrillation, n (%) | 13 (2.7%) | 13 (7.1%) | 0.009 |
| Systolic blood pressure (mmHg) | 118.04 ± 25.53 | 123.43 ± 23.17 | 0.010 |
| Diastolic blood pressure (mmHg) | 70.38 ± 14.60 | 73.63 ± 13.19 | 0.006 |
| Heart rate, beat per min | 90.37 ± 22.32 | 80.16 ± 15.77 | <0.001 |
| Killip class ≥II, n(%) | 129 (70.9%) | 136 (28.5%) | <0.001 |
| White blood cell | 14.13 ± 4.71 | 11.11 ± 3.44 | <0.001 |
| Total cholesterol (mmol/L) | 4.51 ± 1.31 | 4.86 ± 1.27 | 0.002 |
| LDL-C (mmol/L) | 2.74 ± 1.05 | 3.10 ± 1.03 | <0.001 |
| HDL-C (mmol/L) | 0.99 ± 0.24 | 0.97 ± 0.32 | 0.528 |
| Anemia, n (%) | 169 (35.4%) | 105 (57.7%) | <0.001 |
| HbA1c (%) | 6.83 ± 1.63 | 6.56 ± 1.52 | 0.074 |
| Serum albumin (g/L) | 30.48 ± 5.02 | 33.57 ± 4.35 | <0.001 |
| Hemoglobin (g/L) | 121.69 ± 21.86 | 130.63 ± 25.46 | <0.001 |
| eGFR (mL/min/1.73m^2^) | 56.28 ± 32.27 | 85.57 ± 31.14 | <0.001 |
| Serum creatinine (mg/dL) | 1.85 ± 1.56 | 1.14 ± 1.06 | <0.001 |
| LVEF (%) | 46.21 ± 12.31 | 51.75 ± 11.16 | <0.001 |
| Aspirin, n(%) | 168 (92.3%) | 456 (95.6%) | 0.092 |
| Clopidogrel, n(%) | 175 (96.2%) | 457 (95.8%) | 0.841 |
| Statins, n(%) | 173 (95.1%) | 464 (97.3%) | 0.156 |
| ACEI, n(%) | 119 (65.4%) | 360 (75.5%) | 0.009 |
| ARB, n(%) | 34 (18.7%) | 72 (15.1%) | 0.262 |
| CCB, n(%) | 27 (14.8%) | 50 (10.5%) | 0.120 |
| Radial access, n(%) | 95 (64.6%) | 391 (87.7%) | <0.001 |
| Stents, median(Q25~Q75) | 1.00 (1.00~2.00) | 1.00 (1.00~2.00) | 0.200 |
| Contrast volume ≥100 ml, n(%) | 99 (68.8%) | 317 (73.9%) | 0.231 |
| Multi-lesion, n(%) | 112 (76.2%) | 292 (65.5%) | 0.016 |
| Length of hospitalization, median(Q25~Q75) | 6 (5~8) | 12 (8~28) | <0.001 |

Abbreviation: COPD: chronic obstructive pulmonary disease; LDL-C: low-density lipoprotein cholesterol; HDL-C: high density lipoprotein cholesterol; HbA1c: hemoglobin A1c; eGFR: estimated glomerular filtration rate; LVEF: left ventricular ejection fraction; ACEI/ARB: Angiotensin-Converting Enzyme Inhibitors/ Angiotensin receptor blocker; CCB, calcium channel blockers;

**Table S2. Subtypes of infection according to NT-proBNP tertiles**

| Variables | NT-proBNP tertiles | | | |
| --- | --- | --- | --- | --- |
|  | T1 (n=219) | T2 (n=220) | T3 (n=220) | P-value |
| Infection, n(%) | 23 | 39 | 120 | 0.429 |
| Pulmonary infection | 17 (73.9%) | 29 (74.4%) | 96 (80.0%) |  |
| Urinary infection | 2 (8.7%) | 7 (17.9%) | 16 (13.3%) |  |
| Other infection | 4 (17.4%) | 3 (7.7%) | 8 (6.7%) |  |
